# Indication Variability of the Particulate Matter Sensors Dependent on Their Location

**DOI:** 10.3390/s24051683

**Published:** 2024-03-05

**Authors:** Alicja Wiora, Józef Wiora, Jerzy Kasprzyk

**Affiliations:** Department of Measurements and Control Systems, Silesian University of Technology, ul. Akademicka 16, 44-100 Gliwice, Poland; alicja.wiora@polsl.pl (A.W.); jozef.wiora@polsl.pl (J.W.)

**Keywords:** PM_2.5_, PM_10_, measurement network, smog, smoke

## Abstract

Particulate matter (PM) suspended in the air significantly impacts human health. Those of anthropogenic origin are particularly hazardous. Poland is one of the countries where the air quality during the heating season is the worst in Europe. Air quality in small towns and villages far from state monitoring stations is often much worse than in larger cities where they are located. Their residents inhale the air containing smoke produced mainly by coal-fired stoves. In the frame of this project, an air quality monitoring network was built. It comprises low-cost PMS7003 PM sensors and ESP8266 microcontrollers with integrated Wi-Fi communication modules. This article presents research results on the influence of the PM sensor location on their indications. It has been shown that the indications from sensors several dozen meters away from each other can differ by up to tenfold, depending on weather conditions and the source of smoke. Therefore, measurements performed by a network of sensors, even of worse quality, are much more representative than those conducted in one spot. The results also indicated the method of detecting a sudden increase in air pollutants. In the case of smokiness, the difference between the mean and median indications of the PM sensor increases even up to 400 µg/m^3^ over a 5 min time window. Information from this comparison suggests a sudden deterioration in air quality and can allow for quick intervention to protect people’s health. This method can be used in protection systems where fast detection of anomalies is necessary.

## 1. Introduction

Air pollution, and especially its harmful effects on human health, is a problem widely described in the literature. Among the pollutants listed by the World Health Organization (WHO), particulate matter (PM) floating in the air deserves special attention. The most commonly monitored are particulates with a diameter of less than 10 µm, called PM_10_, and particulates with a diameter of up to 2.5 µm, called PM_2.5_. WHO has acknowledged the importance of these pollutants. In the WHO guidelines, the permissible concentrations of PM and other harmful substances in the air have been significantly lowered [1].

Certified state air quality monitoring stations in many countries conduct measurements of the PMs. The results obtained by the stations have high quality. Their geographical distribution is, however, rare. It causes the local concentration to deviate significantly from those provided by the stations. This paper aims to show how much PM concentration can vary by changing the measurement place. It concentrates on PM_10_, but PM_2.5_ is also considered.

### 1.1. Health Impact of PM

PM of anthropogenic origin is the most harmful to health [1]. Its composition is heterogeneous and depends on the type of emission sources and the time it stays in the atmosphere [2]. PM_2.5_ is mainly of anthropological origin [3]. The primary sources of PM are home heating [2,4], industry [5,6], communication [7], including vehicle exhaust and road surface wear [8,9], port activity [10], agriculture (fertilization, plant cultivation) [11], and natural and artificial fires [12]. The main contributors of atmospheric aerosol of anthropogenic origin are carbon compounds. They most often occur as elementary carbon and organic carbon [13]. One of the most carcinogenic are polycyclic aromatic compounds [6,14]. Next are heavy metals such as Cd, Sb, Cr, Cu, Fe, Mn, Ni, Pb, As, Zn, and ionic forms of inorganic compounds [7,15]. The PM oxidative potential (a health exposure metric) increases with the increase in the concentration of the abovementioned chemical compounds [16]. The need to monitor the level of suspended particulate matter appeared together with the knowledge about the harmfulness of these pollutants to human health [17].

The negative impact of air pollution on human health is indisputable. It has been shown that the smaller the particulate matter, the more hazardous it is [1]. The medical literature reveals lung cancer [18,19], chronic obstructive pulmonary disease [20,21], and other conditions [1,22,23] to be caused by carcinogenic compounds, heavy metals, benzo(α)pyrene, and black carbon stemming from the combustion of, i.e., coal in stoves.

### 1.2. Seasonal and Geographical Changes in PM Concentration

Many factors influence the concentration of PM in a given area. The most important are weather conditions. Extended research regarding the region where this investigation was conducted is published elsewhere [24]. PM depends on the seasons and related parameters. The lower the temperature, the higher the concentration of PM, or inversely for areas closer to the equator. Similarly, higher PM is observed with increasing pressure, and the higher the humidity, the higher the PM. Wind strength and direction are of great importance. The weaker the wind force, the higher the PM [25,26]. In Poland, as in other countries, the direction of the wind affects air quality. In summer, PM increases when it blows from the south (Sahara), and in winter, PM decreases when it blows from the west (Scandinavian Lowlands) [25]. In countries where activities for a clean environment are carried out, increases in air pollution caused by blowing PM from other areas are observed [27]. Precipitation reduces PM, especially in autumn and winter, but is less critical than other weather indicators [28]. When significant exceedances of PM concentrations in the air occur, residents are advised to stay indoors. Indoor PM concentrations are about 10–40% of outdoor PM values. However, this is only the case if windows are closed because otherwise, the difference in indoor and outdoor PM concentrations is insignificant [29].

The land layout is no less critical for air pollution. First of all, the density of buildings adversely affects air quality [30]. It has also been observed that on flat terrain, it is only sometimes possible to determine the influence of location on PM concentration. It depends mainly on the number of pollution sources in this area. Meteorological conditions have a more significant effect here [27]. In the case of valleys surrounded by mountains, smog can be retained and accumulated. This happens, for example, in Kraków (Cracow), Poland [31,32], where coal and wood stoves are prohibited. The results confirm that eliminating coal and wood as fuel burned in the home heating has significantly improved the air quality in the city. Nevertheless, it is still one of the most polluted because pollution from areas where such a ban does not apply accumulates above the town. It has been observed that the slower the airflow, the higher the PM concentration [33]. At the same time, it was noted that PM concentrations are lower in coastal areas and higher inland [34].

Improvement of air quality can occur thanks to regional policy [2,16], including the development of renewable energy [35], standardization of measurements, and introduction of air quality guidelines [1]. An unexpected positive effect of the outbreak of the COVID-19 pandemic was the reduction in the concentration of particulate matter in the air. It occurred because of a significant decrease in the economic activity of many enterprises. The lockdown caused a considerable drop in air pollution in Poland by as much as 1/3 compared to 2019 [36].

### 1.3. Methods of PM Measurement

Measurements of particulate matter in the air can be conducted using several methods. The gravimetric method is used as the reference. It is recognized worldwide as the most accurate for PM level in the air. This method was recommended in the European Union by “Directive 2008/50/EC of the European Parliament and the Council of 21 May 2008 on ambient air quality and cleaner air for Europe”. For continuous air quality monitoring, automatic methods with demonstrated equivalence to the gravimetric method and appropriate certification can be used.

Other methods are usually used in information systems, but their indications do not trigger any administrative actions. Many such sensors and measuring systems are designed to measure PM. Although they do not guarantee the correctness of the results, their quality is improving [37,38,39]. One of the main goals of the low-cost sensor (LCS)-based networks has become civil protection. These networks detect the pollution sources, raise awareness on the causes of air pollution, monitor indoor air quality, and are used for personal protection, e.g., students in schools [40].

### 1.4. Measurement Systems Relying on Low-Cost Sensors

A low-cost sensor (LCS) provides the possibility of building affordable wireless and wired measurement networks. LCSs scattered around the city give equally helpful information, though slightly different, as per one certified sensor [41]. For PM measurements, they have been successfully used to determine dust quality in power plant cooling towers [5]. Together with other sensors, they can be components of portable or stationary multisensor platforms for measuring PM and CO, NO_2_, O_3_, SO_2_, CO_2_ gases, and volatile hydrocarbons (e.g., benzene) [42]. It is also possible to measure PM by indirect methods using artificial neural networks based on measurements of NO_2_, temperature, humidity, and wind speed [43]. Information on PM can also be obtained through satellite measurements [44] or by using a fusion of data from LCSs placed on cars and stationary stations. The latter method was used to model the distribution of pollutants [45].

The advantage of using LCSs for measuring PM is their relatively good properties compared to the price. Currently produced sensors give results similar to the reference method [46] and are better than older generation sensors [37]. The quality of measurement systems/sensors depends on the manufacturer [47]. PM sensors are sensitive to interference. Their indications can depend on the type of measured pollution [48]. Humidity is the most common interference [46,47]. The reason is the condensation of water vapor on the particles and the increase in their volume [49]. Sensor manufacturers recommend avoiding fumes, as they significantly overestimate the indications compared to the reference method [41]. Signal drifts may also occur, especially in long-term measurements; therefore, calibration of the sensors is recommended [42,50]. It is also noted that PM_10_ is more challenging to measure with LCSs than PM_2.5_ [51].

### 1.5. Importance of Calibration

Calibration is the activity establishing the relationship between the values of the measurand indicated by any tested instrument and the corresponding values of physical quantities. The most popular are one-, two-, or multipoint calibrations of sensors [52]. If a sensor responds towards several quantities, the calibration process becomes complicated and the number of measurement points skyrockets. Simultaneous changes of a couple of quantities can lower the number of calibration experiments [53]. Bearing in mind that calibration experiments take up time and incur costs, a single-point calibration is often applied [54,55]. Fitting a model to sensor behavior requires many more experiments than during typical calibration. Automation facilitates the process in exchange for the higher cost of instruments used [56]. In some fields, the term ‘calibration’ is sometimes replaced by ‘estimation of model parameters’ based on experimental data but, indeed, it refers to the same activities [57].

Results provided by reference methods compared with responses of LCSs show that the LCSs can be reliable, although they are subject to higher errors. Such measurements, conducted with calibrated sensors placed on drones and a secondary sensor near the reference one on the ground, result in 1–2% errors [58]. Taking air humidity and temperature into account significantly improves measurement quality [54,59]. The influence of these factors can be reduced by applying so-called corrections [60,61].

The measurement result cannot be established without calibration because of the inability to fix the relation with the reference value. The information provided then by such sensors, known in metrology as an indication [62], is still valuable. It can inform one about variability and trends [63]. Within the frame of this work, no formal calibration of PM sensors was conducted. Therefore, the commonly used term ‘result’ was replaced by the metrological term ‘indication’ as more suitable in this circumstance. Typically, measurements end by providing the results, which include estimated values and information about their quality expressed as uncertainty. In this work, complete uncertainty assessment is impossible due to the lack of comparison with the reference.

### 1.6. Citizen Protection

Information on PM provided by measurement networks is primarily used to predict the risks of these pollutants to protect the population [64]. The methods used include machine learning [65], data mining, deep learning [66], artificial neural networks [67], or fuzzy logic [68]. The input data are current and past concentrations of pollutants, current and past weather data, weather forecasts, and the specificity of a given terrain.

National laws and international standards help in efficient human protection. Guidelines for distributing the PM sensor network, their location, quantity, and parameters can be found in the law in force in a given country. In Poland, these are Directive 2008/50/EC and regulations of the Ministry of the Environment. As a rule, they are placed in cities because of their large populations. Localities with a low population several dozen kilometers away from such stations also need to receive information about smog hazards. Due to the limited range of state monitoring systems, the information may be inadequate. In cities, buildings are mainly heated electrically, have gas stoves, or use heat provided by heating plants. In villages far from city centers, ecological methods of heating apartments are usually limited to geothermal energy and heat pumps. Without state subsidies, these systems are expensive and often insufficient during the heating season. Therefore, the most popular method of heating houses in Polish villages is still burning coal and wood. As a result, smoke in winter is a common phenomenon there. Although smoke is still common in rural areas, there are areas being observed where it is decreasing. The effectiveness of some local governments’ actions aimed at improving air quality is visible. The sensor network also made it possible to identify the reasons for the continued exceedances of allowed PM levels.

### 1.7. Motivation for This Work

The main problem to be solved in this article is to examine the impact of the location of low-cost PM sensors on their indications. Previous observations have shown that the quality of air entering the human lungs depends on weather conditions and the activity of air-polluting sources. The more polluted the air, the more negative the impact on human health. When smoke occurs, our health is particularly at risk. To detect a sudden deterioration in air quality, PM LCSs can be successfully used, although they provide poorer-quality information about this pollution. Smoke may appear only in a small area. In unfavorable weather conditions, an incorrectly positioned sensor may fail to detect a threat.

This work aims to solve two main issues. The first one is determining the difference of values provided by instruments measuring PM located at different places but not far away from each other. The second is to determine how to detect rapid changes in the concentration of a substance in a numerical way. This method is described based on data collected from the PM sensor at which smoke occurs suddenly.

## 2. Material and Methods

### 2.1. Place of Investigations

**Figure 1:**
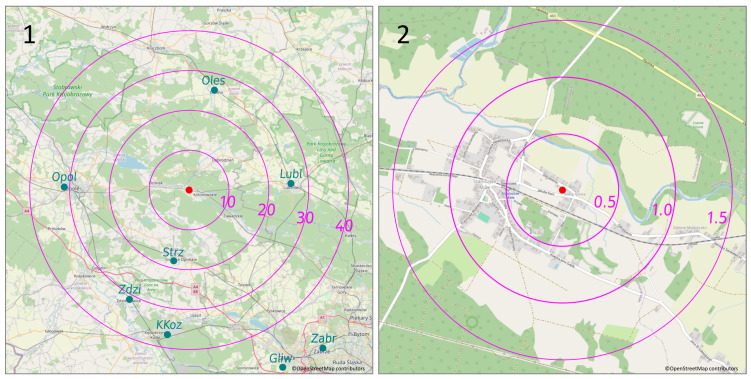
The research location (red dot). **Panel 1** shows the state monitoring stations (olive dots)—current state for 2021. **Panel 2** shows green areas and roads. Circles represent the 10, 20, 30, and 40 km radii for **Panel 1** as well as 0.5, 1.0, and 1.5 km radii for **Panel 2**.

This research was conducted in the village of Staniszcze Małe, Poland, surrounded by forests. Geographical coordinates: 50°39′51″ N 18°19′32″ E, 190 m a.s.l. The terrain is flat. Within a radius of 20 km, the height above sea level ranges from 170 to 260 m. Data were collected during the heating season between January and April 2021, during the ongoing lockdown caused by the COVID-19 pandemic. Meteorological conditions, applicable during the tests, are collected in the report [69]. Figure 8(2) presents mean daily values of wind speed, temperature, pressure at the station level, and relative humidity (RH) registered by the state station in Opole (https://dane.imgw.pl/data/dane_pomiarowo_obserwacyjne/dane_meteorologiczne/dobowe/synop/2021/ (accessed on 17 February 2024)). The nearest state air quality monitoring station is almost 20 km from the research location; others are even further away—see the map in Figure 1(1). Therefore, none of the air quality information provided by these stations covers the study area. The village is disconnected from the gas network; heat pumps and photovoltaic installations are scarce. In the vicinity, there is no industry or main road. The primary heating sources in winter are eco-pea coal or fuel boilers of various origins characterized by low efficiency. The above shows that the primary sources of contamination are the home heating stoves.

**Figure 2:**
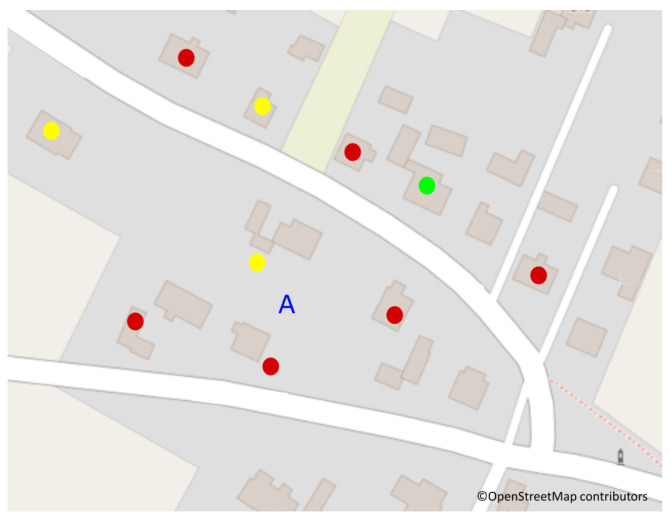
Location A, where the research was conducted, surrounded by the neighbors’ chimneys for eco-pea coal stoves (yellow dots) and coal stoves (red dots). One chimney was not used (green dot).

The research was carried out on a plot of 2500 m^2^, on which there is a detached house. Other detached houses with different types of heating surround this property. The arrangement of smoke sources is as shown in Figure 2.

The selection of the number of sensors and their placement on the field was based on the guidelines provided by the regulation of the Polish Ministry of the Environment and the current state of knowledge in the field of air quality measurement. Several basic guidelines are included here:

1. Size of the monitored area—the larger the area, the more sensors;
2. Number of smoke sources—the more sources, the more sensors;
3. Distance and direction of the smoke source from the measurement place—the farther from the smoke source, the fewer sensors;
4. Distance from buildings and other obstacles taller than a human—the closer the smoke source is, the more sensors are required;
5. Access to power and the Internet;
6. The frequency of indication readings together with processing carried out on raw data, e.g., filtration, interference compensation, influence correction of atmospheric conditions; averaging over a few-minute time window—the closer the source of smoke, the shorter the time window.

In areas other than those studied, there may be area-specific requirements that are not listed here.

### 2.2. Measurement Network

**Figure 3:**
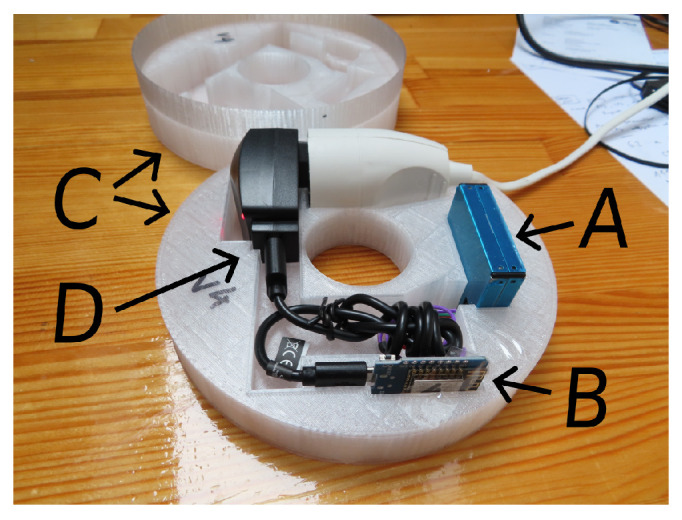
Particulate matter sensor PMS7003 (A) and microcontroller ESP8266 (B) with power supply (D) in case (C).

The network for measuring particulate matter consisted of 12 devices, which included the laser scattering Plantower PMS7003 suspended PM sensor (Figure 3). The working principle and sensor properties of the nearly identical PMS5003 sensor are described elsewhere [70]. The manufacturer recommends using it for indoor measurements at temperatures ranging from −10 °C to +60 °C and relative humidity from 0 to 99%. PM sensors are sensitive to smoke, which strongly contaminates the measuring chamber of optical sensors. Therefore, they should be located away from smoke sources.

Climatic conditions in the study area meet these recommendations almost all year round. The sensor is designed for particle measurements: 0.3–1.0 µm; 1.0–2.5 µm; 2.5–10 μm. The producer provided the accuracy only for particles of 2.5 µm. It usually means that for other particle classes, the accuracy is worse. Despite these limitations, this sensor has linear characteristics, high reproducibility, and low sensitivity to humidity among low-cost PM sensors operating outside the building [47,49].

**Figure 4:**
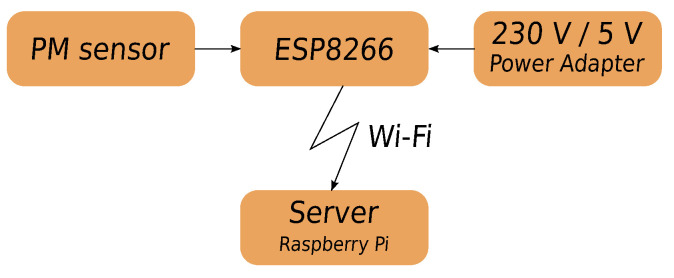
Connection diagram of the measuring system elements.

**Figure 5:**
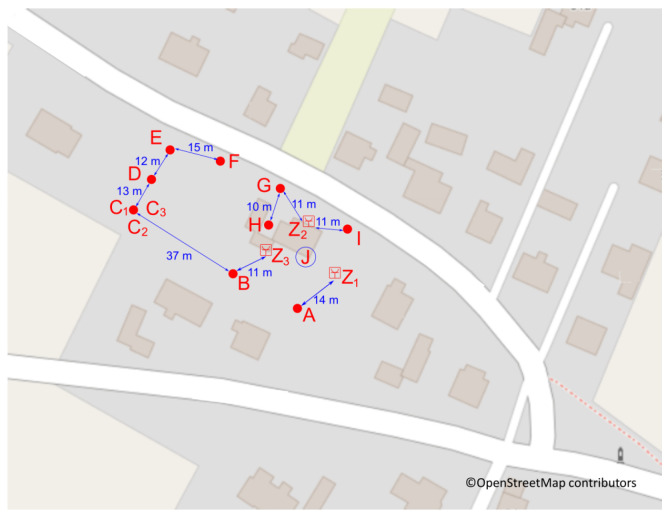
The networkfor measuring particulate matter: (A–J)—Location A–Location J of the sensors; (Z)—power supply of the measuring network.

The ESP8266 microcontroller, intended for home automation solutions, equipped with a Wi-Fi communication module, collected measurement data and transmitted them to a server (Figure 4). The sampling rate was about 1 per second. The microcontroller was only responsible for reading data from the sensor and transmitting them to the server. A battery power supply for the measuring system was considered. Still, considering that battery efficiency significantly decreases at sub-zero temperatures, it was decided to power it from the grid. Figure 5 shows a map of the measuring plot and the location of the sensors constituting the measurement network. The sensors were located in accordance with the regulations of the Ministry of the Environment for PM measurements in microscales. The sensors were labeled A through J. Sensors C1–C3 were placed at one point to check the reproducibility of the measurements. Sensor J was inside the building. A weather station was set to control humidity, temperature, wind strength, and direction of the farthest from obstacles.

The data most presented here are the means calculated over a 5 min time window. The window width was selected based on tests not included here as a compromise between the number of samples (about 300) and the time elapsed. Averaging over 300 samples significantly decreases the uncertainty contribution evaluated using the Type A method by about 17 times. A longer time window makes the plots smoother but hides information about short-term changes.

### 2.3. Phases of Experiments

**Figure 6:**
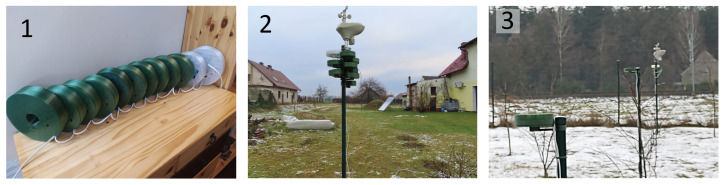
PM sensors at the different research conditions. (**1**) Condition a: Tests of the sensors in the building; (**2**) Condition b: Measurements with Sensors A–J at Location A; (**3**) Condition c: Measurements with Sensors C1–C3, D, and E at Locations C, D, and E (other sensors not shown in the photo).

The experiments were carried out according to the schedule presented in Table 1. Figure 6 shows the sensors at these three research conditions. The condition change was targeted to investigate the influence of the location on the PM indications.

## 3. Results and Discussion

The few-weeks-long experiments focused on two fundamental questions: (1) whether the location of the sensor affects PM measurements, and (2) how to recognize the moment when smoke begins to appear in the air.

### 3.1. Effect of Location on PM Measurements

The main problem that had to be solved was checking whether the sensor indications were independent of their location in a relatively small area. Organoleptic experiments suggest that such a relationship exists, although it has not been demonstrated numerically so far. It can be verified only if the built measurement network meets the basic metrological requirements. It was not necessary to check the sensors against the reference method because the variations between indications at different locations were important. From the human health point of view, much more critical are changes in indications of PM values. These changes allow the determination of the rapid change in air quality. State air quality monitoring stations provide data with an hour interval. In the case of this research sensor network, information about sudden smokiness may appear within minutes. To verify the concept, the research was conducted in winter, from January to April 2021.

#### 3.1.1. Initial Tests of the Measurement Network

**Figure 7:**
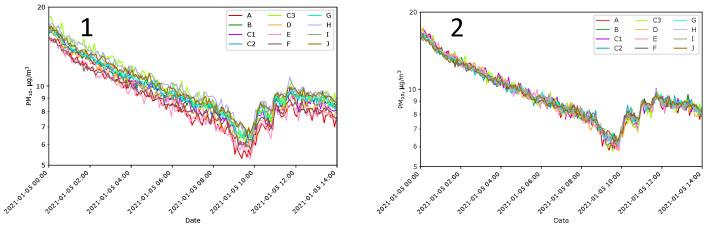
Sensor gain correction. PM_10_ indications of Sensors A–J before the gain correction (**Panel 1**) and after this correction (**Panel 2**).

The first step was to test all the sensors in a room at Location J at temperature of approximately 21 °C and constant humidity (Figure 7(1)). It can be observed that the signals from the sensors are shifted nearly parallel in the log scale. It suggested that the problem is incorrect gains. Therefore, we decided to recalculate the acquired data so that, for the day when the sensors were in the room, all indications became similar. The recalculated *i*-th indication $X_{ik}^{\prime }$ of sensor *k* was

(1) $$X_{ik}^{\prime }=\frac{\frac{1}{12}\sum_{\kappa =A}^{\kappa =J}\left(\frac{1}{N_{\kappa }}\sum_{\iota =1}^{N_{\kappa }}X_{\iota \kappa }\right)}{\frac{1}{N_{k}}\sum_{\iota =1}^{N_{k}}X_{\iota k}}X_{ik}$$

where $X_{ik}$ is the *i*-th indication acquired from sensor *k*; k ∈ {A, B, C1, C2, C3, D, E, F, G, H, I, J}, and $N_{k}$ is number of indications accounted during this normalization related to sensor *k*. After recalculations, the indications from all PM_10_ sensors, expressed in µg/m^3^, became much more similar (Figure 7(2)).

**Figure 8:**
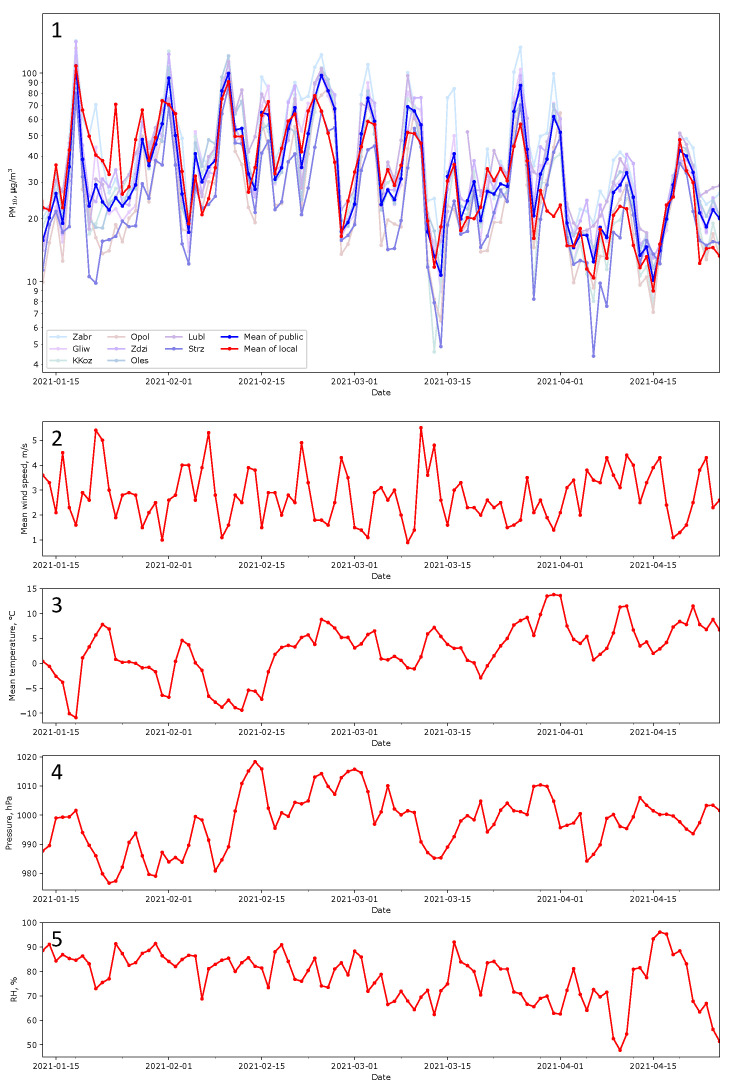
Comparison of PM_10_ indications performed by the elaborated system with the state stations (**Panel 1**) and meteorological conditions (**Panels 2**–**5**). On **Panel 1**, the mean of indications (red line) provided by Sensors A–J is compared with the mean of the state data gathered in 60 km radii (blue line) and measurement values provided by these state stations (light lines). Labels for the stations are in line with Figure 1. **Panels 2**–**5** present mean daily values of meteorological quantities registered by the state station in Opole.

Comparing indications of LCSs with the reference method was not possible. It was also not the most important. The purpose was to demonstrate differences in sensor indications, not their measurement values. The comparison of data obtained from the measurement network with data obtained from state monitoring stations was made after the entire experiment. The task cannot be named ‘calibration’; it was, rather, ‘fitting’ of the data to be comparable with results obtained by state stations. The nearest seven state stations were in a straight line from 20 km to 60 km from the experiment site in different geographical directions. Their data were used as the reference. Figure 8(1) shows the corrected indications from the local sensors compared to the data provided by the state stations. The time window used for averaging was one day. The correction factor of 0.4 applied for all local indications gathered during the experiments allows for fitting to the values obtained by the state stations. All data presented in this work use this factor.

#### 3.1.2. PM Measurements at One Location

**Figure 9:**
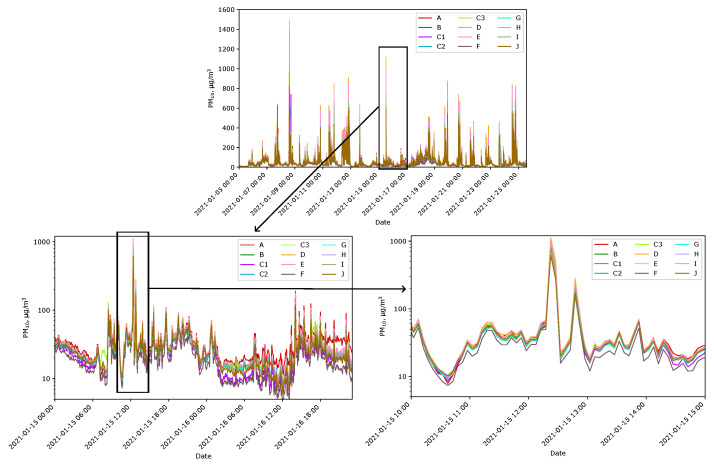
PM_10_ indications of Sensors A–J gathered at Location A and magnifications of selected periods.

The placement of all the sensors at Location A was to determine how much the indications of suspended PM would differ between the sensors. Figure 9 shows the indications for PM_10_ obtained from the sensors during two January weeks at Location A. For PM_2.5_, the nature of the responses was very similar. Periodic increases in the PM level were caused by the activation of old stoves heating some neighbors’ buildings. The nearest active chimney was located approximately 25 m south of Location A.

The data obtained from the measurement network confirmed the importance of choosing the sensor location. At periods when all sensors were at Location A and virtually no chimney was active, e.g., at 2021-01-15 3:00, the maximum differences in indications between all installed sensors were 10 µg/m^3^ and 8 µg/m^3^ for PM_10_ and PM_2.5_, respectively. The mean indications then were PM_10_ = 25 µg/m^3^ and PM_2.5_ = 19 µg/m^3^. When the smoke caused by operating chimneys was significant, e.g., at 2021-01-15 12:22, these maximum differences increased to 450 µg/m^3^ and 220 µg/m^3^, respectively. The mean indications then were PM_10_ = 780 µg/m^3^ and PM_2.5_ = 430 µg/m^3^.

**Figure 10:**
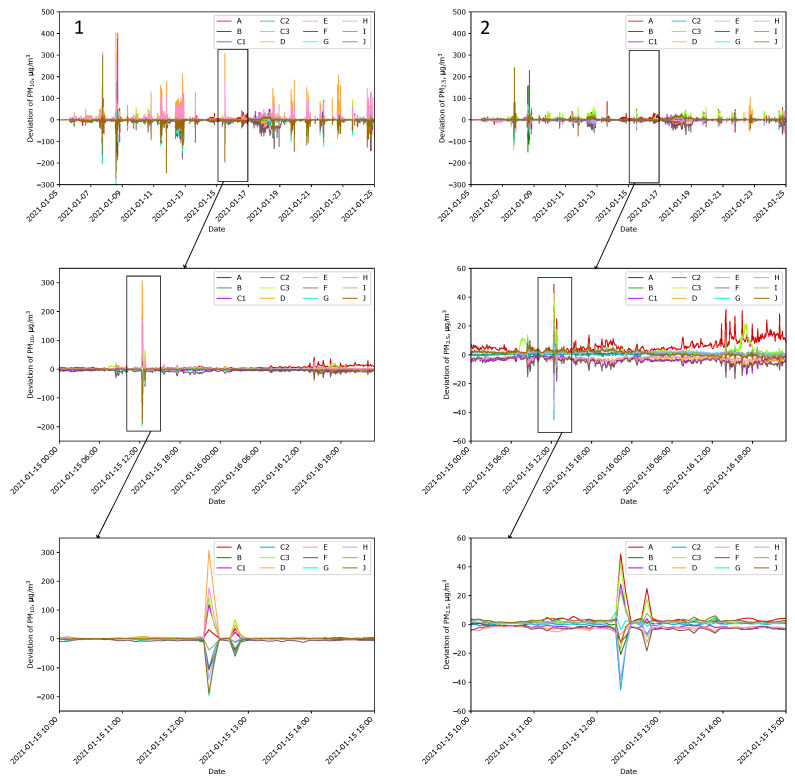
Deviation from mean indication of Sensors A–J measuring PM_10_ (**Panel 1**) and PM_2.5_ (**Panel 2**) at Location A. The first row presents a 20-day interval in January, while magnifications are in the following rows. The magnified periods are the same as in Figure 9. Notice the change in the y-axis scale.

Figure 10 shows the deviation ($\Delta_{i}$) of indications for PM_10_ and PM_2.5_ in the initial phase outside the building (Condition b) at Location A. The deviation is calculated as the difference between the indication and the mean of all outdoor Sensors A–I:

(2) $$\Delta_{i}=X_{i}-\frac{1}{11}\sum_{j=A}^{j=I}X_{j}$$

where $X_{i}$ and $X_{j}$ are the indication of Sensors *i* and *j*, respectively, where j ∈ {A, B, C1, C2, C3, D, E, F, G, H, I}, and i ∈ {A, …, I, J}. The indications of Sensor J are not included in the mean. It was the only sensor either placed in the house at Location J when other sensors were spread over the plot or it was together with other sensors at Location A. The deviations calculated for the periods with no intense smoke were small, at the level of several or a dozen µg/m^3^. When smoke appeared that could be sensed with the human nose, these deviations increased even to several hundred µg/m^3^. The sudden increase in the differences between the indications can be treated as the first predictor of smoke, containing, i.e., particulate matter.

Some conditions which hindered the measurement occurred during the experiments. Placing twelve sensors on one post at Location A caused the constricted flow of air to these sensors because the inlets were located at the bottom of the housing. In addition, the manufacturer recommends operation in much lower measurement ranges than those recorded during tests. Another recommendation is to avoid direct sensor contact with smoke. The proximity to the chimneys also did not ensure proper mixing of smoke with air. Therefore, all values obtained in smoke conditions are treated only as indicators of the increase or decrease of pollutants. The values are not treated as the “measurement values” in the metrological sense, in line with VIM [62].

#### 3.1.3. PM Measurements in Various Locations

**Figure 11:**
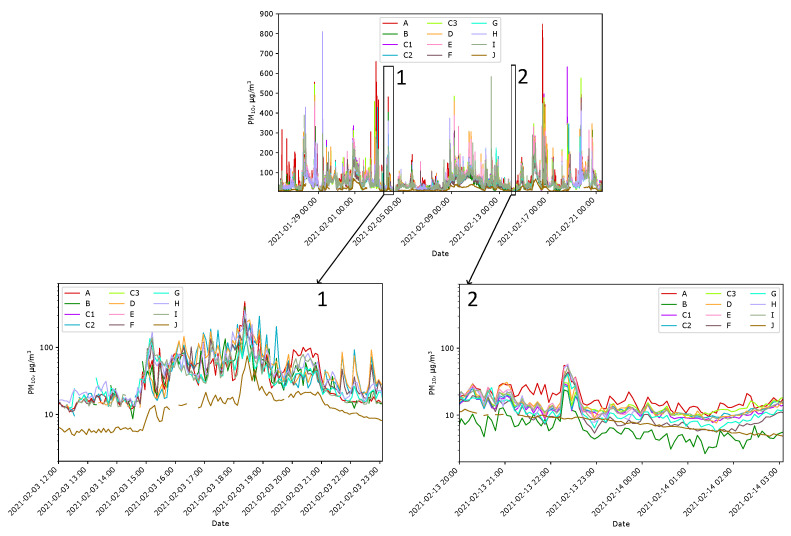
PM_10_ indications of Sensors A–J at Locations A–J in unfavorable weather conditions and smoke (**1**) and in good conditions without smoke (**2**). Note that the magnified plots are in the log scale on the PM axis.

In the next phase of the experiments, the arrangement of the sensors on the plot was in accordance with the schedule shown in Section 2.3. Each sensor was placed following Figure 5. Figure 11 shows graphs illustrating data gathered during approximately four weeks of research. The data confirm that the air quality varies depending on where it is measured, especially in the case of intense smoke near one of them.

##### Differences in Indications over Time

**Figure 12:**
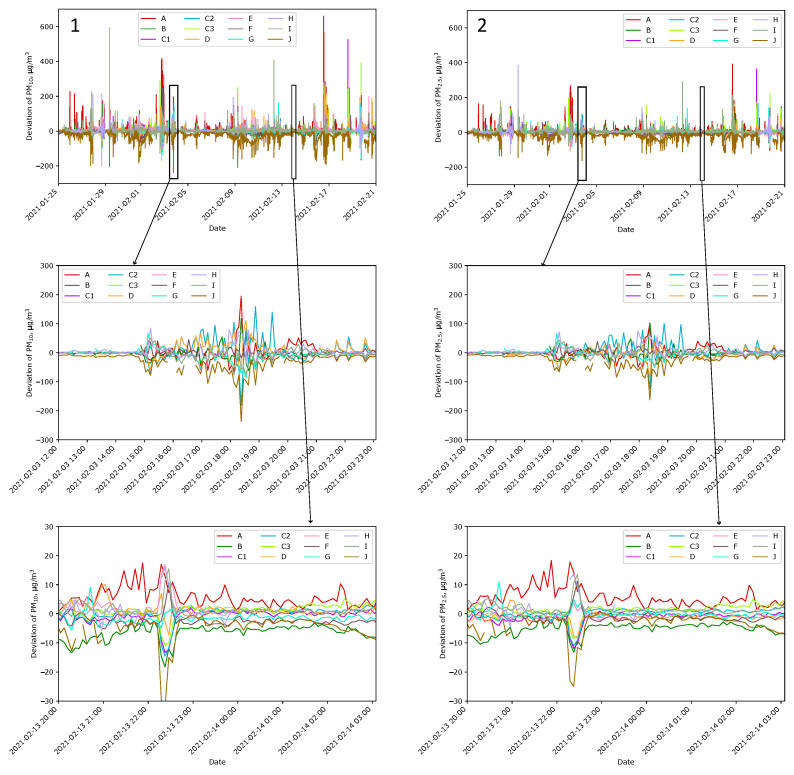
Deviation from mean indication of Sensors A–J measuring PM_10_ (**Panel 1**) and PM_2.5_ (**Panel 2**) at Locations A–J for Sensors A–J. The first row presents a 1-month interval in February, while magnifications are in the following rows. Plots in the second row correspond to the typical smoky conditions. Plots in the third row correspond to the clean air conditions. Note the change in the y-axis scale.

**Figure 13:**
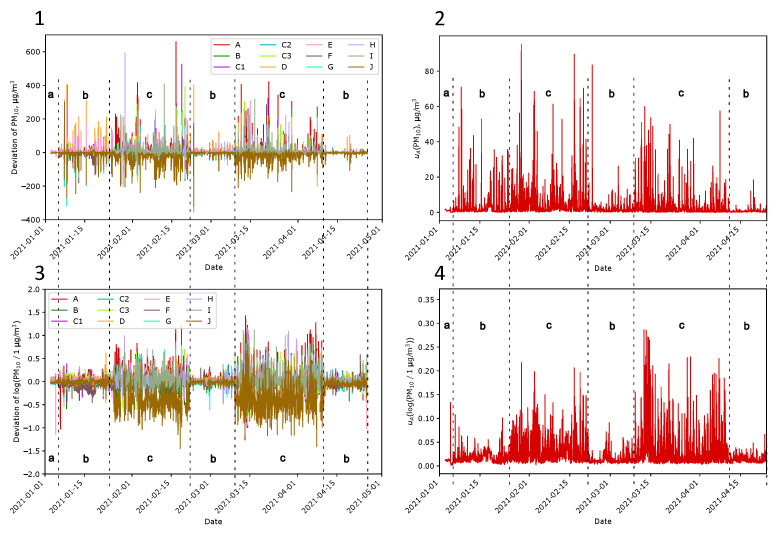
Differences in PM indications over four months. Deviations for Sensors A–I calculated as the difference between the particular indication and the mean over Sensors A–I (**Panel 1**) and the deviations calculated in the log scale according to Equation (5)—(**Panel 3**). Uncertainty contribution calculated using the Type A method based on Sensors A–I expressed in linear scale (**Panel 2**) and in log scale (**Panel 4**). In different periods, Sensors A–J were inside the building (a), outside the building at Location A (b), and spread outside the building at Locations A–J (c). During Period c, Sensor J was indoor, so its indications were lower than those of others.

As earlier, when all sensors were at one location, the deviation of indications from the mean was calculated for PM_10_ and PM_2.5_ for sensors spread at Locations A–J. Figure 12 shows the results. They are similar to those presented in Figure 10, but the dispersions are several times higher. The differences can be better seen in Figure 13(3). The logarithmic deviations ($\Delta_{i}^{\log}$) are presented here as the difference between the logarithm of the indication and the mean over the logarithms of the indications calculated for Sensors A–I:

(3) $$\Delta_{i}^{\log}=\log\frac{X_{i}}{X^{0}}-\frac{1}{11}\sum_{j=A}^{i=I}\log\frac{X_{j}}{X^{0}}$$

where $X^{0}=1$ µg/m^3^. The nature of the indications during the measurements at Location A (Figure 13 periods b) and A–J (Figure 13 periods c) are different. In the case of measurements at one location, the values for all sensors coincide in time. The observed differences arose from the lack of air–smoke mixture homogeneity and the short-term exceeding of the sensor measurement range. In the case of measurements at Locations A–J, the characteristics usually do not overlap so well over time.

Figure 13(2) shows Type A standard uncertainties calculated from all outdoor sensors as follows:

(4) $$u_{A}\left(X\right)=\sqrt{\frac{1}{n}\frac{1}{n-1}\sum_{i=A}^{i=I}\Delta_{i}^{2}}$$

where *n* is the number of outdoor sensors for which data are available. Similarly, Figure 13(4) shows uncertainties for logarithmic data calculated as follows:

(5) $$u_{A}\left(\log\left(\frac{X}{X^{0}}\right)\right)=\sqrt{\frac{1}{n}\frac{1}{n-1}\sum_{i=A}^{i=I}{\left(\Delta_{i}^{\log}\right)}^{2}}$$

It should be emphasizedthat Type A uncertainty does not provide complete information about the quality of measurements. Instead, it informs only about the variety of indications.

Table 2 better illustrates differences in indication varieties. Here, the means of uncertainty over each period are calculated. The uncertainty of PM expressed in linear scale follows this concentration. We observe a relatively high mean of uncertainty in Period #2 because the air was poor. Comparing the neighbor values, the uncertainty is lower when measured at one place. More distinct are the uncertainties of PM expressed in the log scale. For indoor initial measurements, the mean is 0.010; when measured at one spot, it is about 0.014, and when measured spread over the plot, it is about 0.026. Because the observed uncertainty is the square root of the squared uncertainty sources, we can distinguish the following source uncertainties in log scale [71]:

- Sensor accuracy—0.010;
- PM heterogeneity around the post—0.010;
- PM heterogeneity over the plot—0.022.

**Figure 14:**
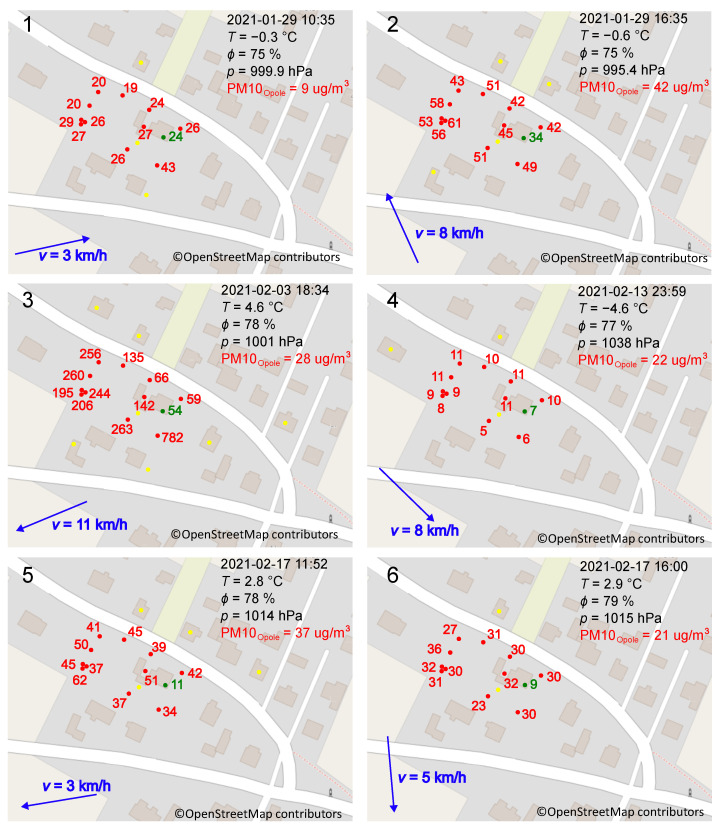
PM_10_ distribution over the plot. **Panel 1**–**Panel 6** illustrate different cases registered during investigations. Dates and times are printed on the top-right corners. The red numbers illustrate PM_10_ indications in µg/m^3^ provided by Sensors A–I at Locations A–I outside the building, while the green ones—inside at Location J. The following meteorological conditions: temperature *T*, relative humidity ϕ, atmospheric pressure *p* in black, PM_10_ in red, and wind strength with direction in blue, are cited after the state monitoring station in Opole. Yellow dots represent working chimneys.

The value of 0.010 in the log scale corresponds to 2.3% relative uncertainty, while 0.022 corresponds to 10% relative uncertainty. The presented values are means of relative standard uncertainties calculated over a long period of time. It means that short-term variations are significantly higher, even over tenfold, as evidenced in Figure 14(3) below.

##### Analysis of Specific Moments

**Figure 15:**
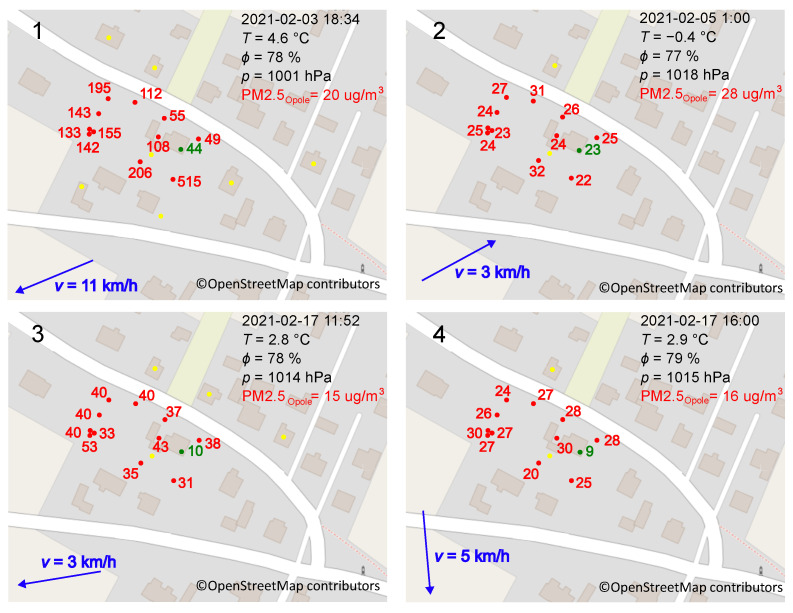
PM_2.5_ distribution over the plot. **Panel 1**–**Panel 4** illustrate different cases registered during investigations. Dates and times are printed on the top-right corners. The red numbers illustrate PM_2.5_ indications in µg/m^3^ provided by Sensors A–I at Locations A–I outside the building, while the green ones—inside at Location J. The following meteorological conditions: temperature *T*, relative humidity ϕ, atmospheric pressure *p* in black, PM_2.5_ in red, and wind strength with direction in blue, are cited after the state monitoring station in Opole. Yellow dots represent working chimneys.

Figure 14 and Figure 15 show examples of PM_10_ and PM_2.5_ maps, respectively, prepared thanks to the elaborated system. Indications provided by the measurement network at Locations A to I (the letters are the same as in Figure 5) were compared with data obtained from the state station in Opole, 30 km away from the research site. These maps present indications for different times of the day, activities of the nearest chimneys, and weather conditions. Observations of PM_10_ and PM_2.5_ indications show the dependence of measurement locations on these indications. In particularly unfavorable conditions, the indication of one sensor may be much higher than another sensor not exposed to increased smoke. An example is the situation shown on the map in Figure 14(3). On 3 February 2021at 18:34, the mean of PM_10_ indications was about 800 µg/m^3^ at Location A, while it was about 60 µg/m^3^ at Locations G and I. Working chimneys surrounded the sensor at Location A. In addition, the wind carries smoke to the sensor from further areas. The indications of Sensors G and I are about 13 times lower. These locations were approximately 20 m and 30 m north of Location A. In contrast to the measurements at Location A, the wind did not carry smoke directly to the sensors at Location G and I.

This over-tenfold difference in indications raises the question of representative places of measurement. Typically, the state stations measure the quantity of interest in one spot using a top-quality sensor. The places are chosen according to international standards, but citizens breathe air in as it is. The above-presented data show that some places can be much worse than others. Therefore, the design of future monitoring stations should be followed by consideration of building a measurement network that allows the sampling of air in many places. It can be realized in a way similar to that presented here.

Maps presented in Figure 14(1,2) and Figure 15(1) confirm the following: Even though the wind does not directly carry pollutants toward it, the sensor placed close to the source of air pollution shows significantly higher indications compared to other sensors. When the stoves are inactive, the indications in the entire study area are similar (Figure 14(4,6)) but not exactly the same. The indications shown in Figure 14(6) are much higher than in Figure 14(4). This is mainly due to the direction and strength of the wind and the time of day. During the day, the residents are more active. Despite the lack of chimney operation in the immediate vicinity, remote buildings increase air pollution. Let us analyze the indications of Sensor H surrounded by buildings on three sides. They show that polluted air accumulates in such spaces and stays there longer due to insufficient ventilation in the spaces between buildings (Figure 14(5) and Figure 15(3)).

**Figure 16:**
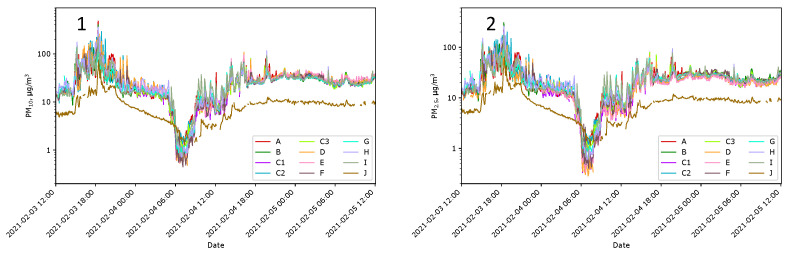
Comparison of the sensor indications inside the building at Location J (golden brown line) with the sensor indications outside the building for PM_10_ (**Panel 1**) and PM_2.5_ (**Panel 2**).

Very high air pollution caused a significant increase in PM_10_ and PM_2.5_ inside the building. The observations showed that the indoor PM_10_ was 10–20% of the outdoor PM_10_, and the indoor PM_2.5_ was about 20–40% of the outdoor PM_2.5_. This is indicated, among others, by data presented in Figure 14, Figure 15 and Figure 16. In unfavorable conditions, such as on 3 February, the value of PM_10_ inside the building was 54 µg/m^3^, while PM_2.5_ was 44 µg/m^3^. The next day, between 5:00 and 8:00, a quick and short-term improvement in outdoor air quality was noted. Due to inertia in air exchange, higher PM values were observed inside the building compared to outside. At that time, the station in Opole did not record measurement data. Another station in Strzelce Opolskie, located to the south of the research site, recorded PM_10_ values from 2.4 µg/m^3^ to 4.8 µg/m^3^ between three and four hours later (wind direction: NW; wind speed: 10–20 km/h). On the night of 13–14 February, under favorable weather conditions, PM_10_ inside the building was also close to that registered outside and amounted to 7 µg/m^3^, while PM_2.5_ amounted to 6 µg/m^3^. In addition, PM_10_ and PM_2.5_ inside the building were similar. It follows that particles with a diameter of less than 2.5 µm entered the building easily through leaks in the windows of the building and ventilation.

In conditions when the chimneys were inactive, PM_10_ and PM_2.5_ fell to values similar to those recorded by the state monitoring stations. In most cases, they did not reach them and were consistently higher primarily due to the fact that ventilating the area out of pollution took up too much time. The stoves were started before the air was cleaned out of impurities, and the atmosphere was smoked again. The data obtained, including those presented in Figure 14 and Figure 15, and the data included in the report [68], show that the wind direction caused the greatest variation in the indications. Other meteorological parameters such as wind, temperature or atmospheric pressure have a lower impact. In the case of easterly, southerly and south-easterly winds, slower air purification was observed than when the winds blew from the north, west or north-west. This is because, to the northwest of the research site, there are large forested areas without large industrial centres. On the contrary, to the southeast, there is the Silesian Agglomeration, characterized by extensive industry and a dense population. Higher air pollution levels result from the transport of these pollutants from highly industrialized and urbanized areas.

**Figure 17:**
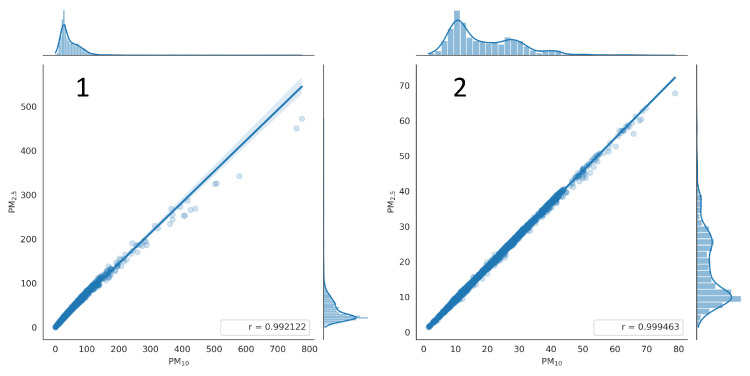
Example of correlation between PM_2.5_ and PM_10_ indications acquired for Sensor A (**Panel 1**) and Sensor J (**Panel 2**) recorded from 1 to 20 February 2021.

The correlation between PM_2.5_ and PM_10_ is presented in Figure 17, for Sensor A—located outdoors (Panel 1) and for Sensor J—located indoors (Panel 2). The data is presented for the period from 1 February to 20 February. The PM_2.5_/PM_10_ ratio for Sensor A is 0.7 and for Sensor J is 0.9. This is because larger particles are easier to filter out, so it is more difficult for them to get into the building. For Sensor J located indoors, the points deviate slightly from a straight line with the increase of PM_10_ concentrations. The mode (the most probable value) for PM_10_ is 10 µg/m^3^. For Sensor A located outdoors, the PM_10_ mode is 30 µg/m^3^, and it visibly deviates from the line above 300 µg/m^3^ when heavy smoke around the sensor occurs.

The discrepancies in the indications intensify with the wind strength and direction. It is also relevant which chimneys are currently working. Overvalues are primarily influenced by smoke coming from the nearest chimneys. The general increase in pollution is also caused by chimneys located further away from the study plot and the background measured by the state stations.

### 3.2. Recognition of Smoke in the Air

**Figure 18:**
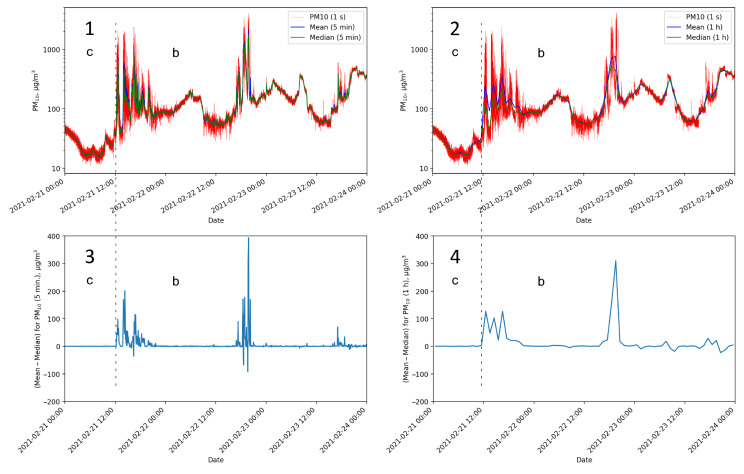
Mean–median comparison for Sensor J. **Panels 1** and **2** present raw signals acquired about every 1 s (red line), mean values (blue line), and medians (green line). The differences between the means and medians are shown in **Panels 3** and **4**. **Panels 1** and **3** use 5 min time windows while **Panels 2** and **4**—1 h time windows. The sensor was at different locations: inside the building at Location J (Period c) and outside the building at Location A (Period b).

The elaborated measurement network allowed sampling with a relatively high frequency (about 1 sample/s), which reveals noise in the signal. Figure 18(1) shows the raw signal obtained from Sensor J and the mean and median values over 5-minute time windows. Sensor J was located inside the building when others were spread over the plot or outside, together with others at Location A. During the experiments, the means and medians were similar when none of the nearby chimneys was working, which made the air composition homogeneous. It happens primarily during periods of the sensor staying in the room or at night at Location A—see Figure 18. When the smoke from the chimney was near the sensor, i.e., when smoke and air were not homogeneous, the means and medians differed. This is especially visible when the indications are substantial and exceed the sensor ranges. This generally happened in the afternoons. High signal values significantly increased the mean. The median was more resistant to such disturbances. This phenomenon was observed for all sensors throughout the study.

Figure 18(3,4) presents the difference between means and medians obtained from 21 February to 23 February 2021. As previously observed, this difference is a valuable indicator of the appearance of smoke in the air, which also affects human health. In the case of minimizing the harmful impact of pollutants on human health, such a distinction can be of great importance. It can be useful, especially in the dense urban area of single-family houses heated with nonecological sources. This indicator can be used as an alarm signal to quickly move away from such a place with a significant increase in smoke.

As shown in Figure 18, in Period c, the difference between the mean and the median is slight and falls within the error declared by the manufacturer. The mean and median values also confirm that the air in the building is the cleanest. Still, even with the windows tightly closed, the increase in pollutant levels can be exceeded in unfavorable circumstances, e.g., on 3 February (golden brown line in Figure 11).

**Figure 19:**
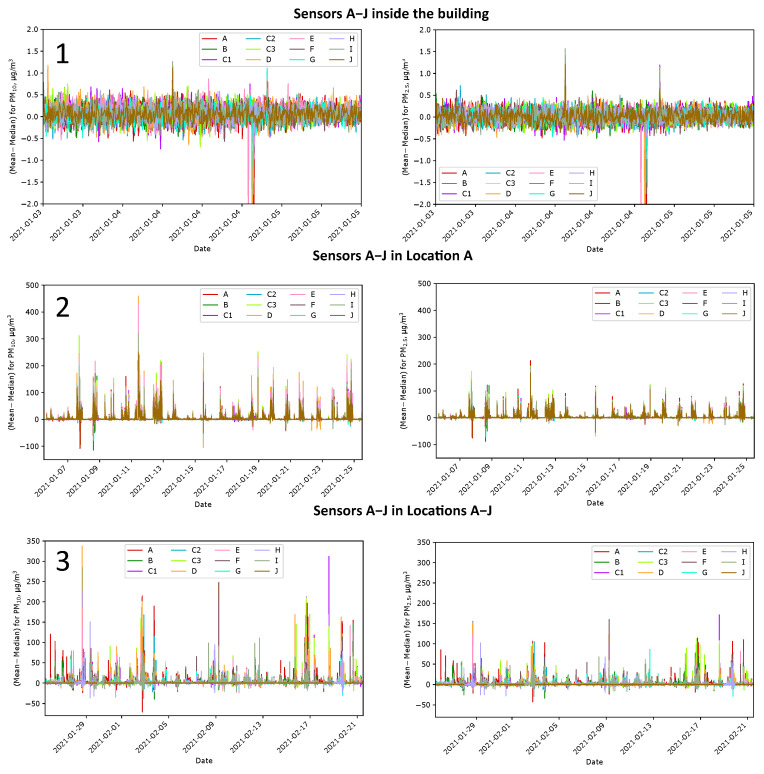
Mean–median differences calculated for PM_10_ and PM_2.5_ at all stages of the research: during the preparation of sensors for operation (**1**), measurements at Location A (**2**), and measurements at Locations A–J (**3**).

The plots in Figure 19 show the differences between the means and medians measured indoor and outdoor at Location A, and outdoor at Locations A–J. The differences in the building without smoke are slight and fit the accuracy limits. However, these differences can increase up to about 300 µg/m^3^ for PM_10_ and up to 150 µg/m^3^ for PM_2.5_ when smoke clouds appear near the sensors. Pollutants in the air are not mixed and do not constitute a homogeneous mixture. The nature of the changes in the mean–median differences at one place (Location A) is other than at different places (Locations A–J). As previously noted, in the case of measurements in one place, the differences between the means and medians are more correlated over time. In the case of measurements at Locations A–J, these differences are more chaotic, as they depend on the source of smoke and weather conditions.

The increase rate of these differences depends on the dynamic properties of the PM sensor. This work chose a 5 min time window to calculate the means and medians. The window size is due to the amount of data, and there is no need to update the data more often. If the differences in means and medians need to be obtained more frequently, the time window can be changed as required.

## 4. Conclusions

The designed and built network of twelve devices measuring PM_2.5_ and PM_10_ was based on the low-cost PMS7003 particulate matter sensor and the ESP8266 microcontroller. The sensors were placed on a small plot of land with a residential building, surrounded by several other single-family houses heated mainly by coal stoves.

The first issue solved in the frame of this work is research on the influence of the sensor location on obtained indications. Studies have shown that indications recorded by these sensors largely depend on the measurement location. The difference in indications can reach more than tenfold at the distance of 20 m. The place of smoke generation, the direction of the wind, and high obstacles on the plot, e.g., buildings, influence the indications. Sensors located closer to the source of the smoke, accounting for the direction of the blowing wind, showed higher values of pollutants. As the literature shows, information about the quality of the air that a person breathes in is undoubtedly important. Monitoring stations located dozens of km away from many smaller towns do not provide enough precise information to their inhabitants. The values provided may be significantly lower than those observed locally, inhaled by residents of particularly unfavorable places.

This is because houses in small towns are often heated by low-efficiency stoves, which pollute the air much more than the heating systems used in large cities. Smoke from low chimneys in unfavorable conditions does not rise upwards but enters the houses of neighboring buildings through the gaps in the windows and air vents. A significant increase in pollutants in such conditions is local and may vary significantly, even on the surface of 2500 m^2^. Therefore, air quality should be monitored closely enough to include rural areas. Additionally, we recommend considering whether a small network spread of cheaper sensors would provide more valuable information than one expensive sensor. It should be conducted within the available funding limits during the design of the monitoring stations.

The second issue considered in this work was the numerical smoke detection. A good predictor is the difference between the mean and the median calculated in a five-minute time window. When there is no direct smokiness, this difference is within a few μg/m^3^. With smoke, these differences increase and reach several hundreds of μg/m^3^. A sudden increase in this difference may cause residents to return immediately to the building and protect against the influx of outside air. Detecting a sudden increase in air pollutants is intuitive because it is based on the experience of many people. This method can be used not only for air quality control but, generally, in protection systems requiring quick detection of anomalies. The novelty of this subject is a simple way to distinguish this phenomenon numerically using low-cost devices.

## Figures and Tables

**Table 1:** Research schedule.

#	Time Period	Condition	Description
1.	3 Jan 2021– 5 Jan 2021	Condition a	Device tests, repeatability, gain
2.	5 Jan 2021–25 Jan 2021	Condition b	Outdoor tests at one Location A
3.	25 Jan 2021–21 Feb 2021	Condition c	Outdoor tests at Locations A–J
4.	21 Feb 2021–9 Mar 2021	Condition b	—
5.	9 Mar 2021–10 Apr 2021	Condition c	—
6.	10 Apr 2021–25 Apr 2021	Condition b	—

**Table 2:** Means of Type A uncertainties in different periods—cf. with Table 1.

#	Description	$u_{A}\left({\mathrm{PM}}_{10}\right),{µg/m}^{3}$	$u_{A}\left(\log\left({\mathrm{PM}}_{10}/1\;{µg/m}^{3}\right)\right)$
1.	a—indoor	0.84	0.010
2.	b—at one spot	2.1	0.016
3.	c—spread	3.4	0.027
4.	b—at one spot	1.2	0.013
5.	c—spread	1.7	0.029
6.	b—at one spot	0.58	0.013

## Data Availability

The maps used to prepare Figure 1, Figure 2, Figure 5, Figure 14 and Figure 15 are available under the Open Database License. Other data are contained within the article.
